# Supplementary material for: Dimensionality reduction of calcium-imaged neuronal population activity
Source: Nat Comput Sci. Author manuscript; Available in PMC 2023 Jul 20. (PMC10358781; doi:10.1038/s43588-022-00390-2)
Supplement: Supp Mats [file NIHMS1905247-supplement-Supp_Mats.pdf]

Supplementary Figures

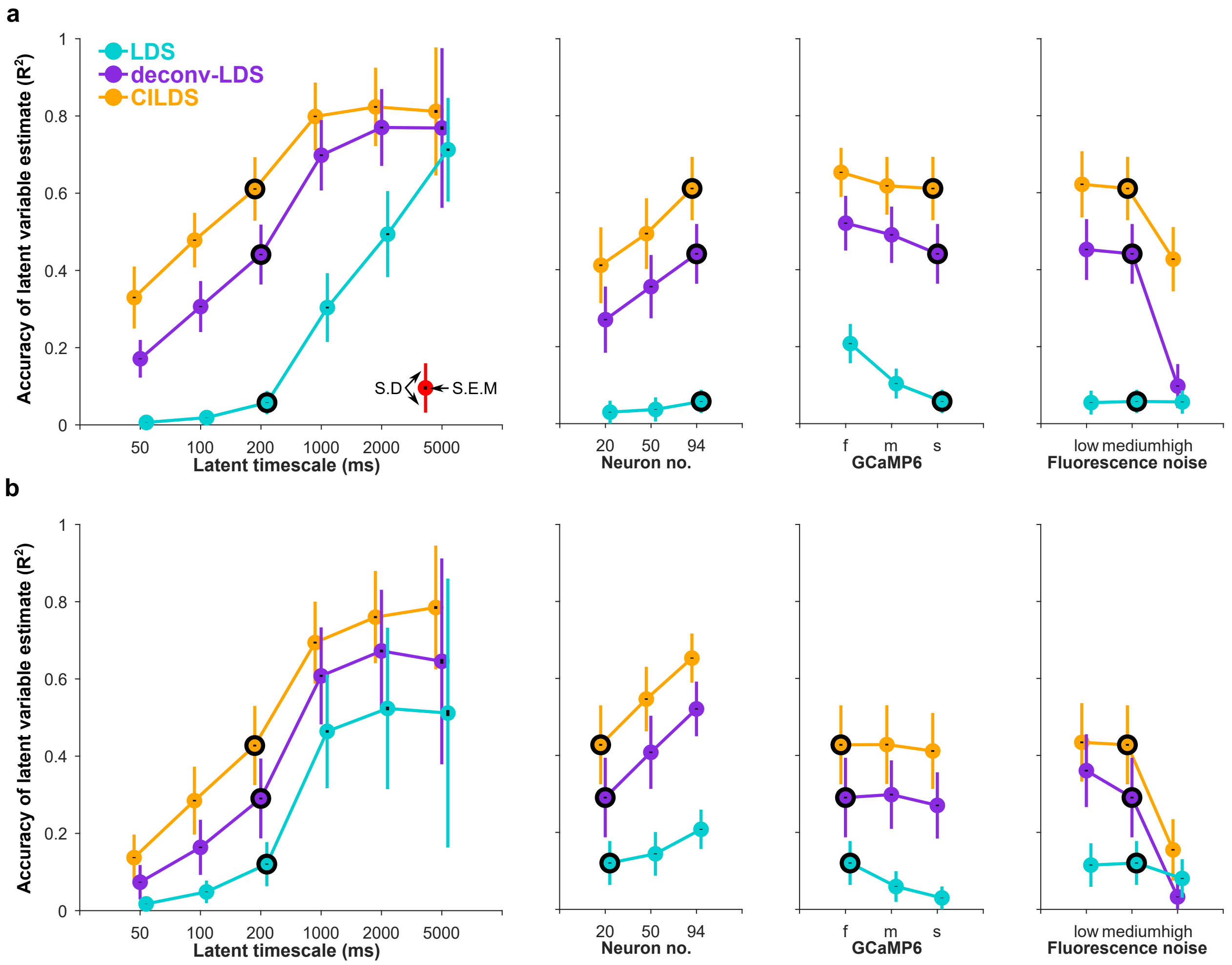

Supplementary Figure 1. Accuracy of latent variable recovery for additional combinations of experimentally-relevant variables. Same conventions as Fig. 3d-g. These experimental variables are different from Fig. 3 in the following ways: **(a)** a calcium decay constant matching the calcium indicator GCaMP6s instead of GCaMP6f and **(b)** a smaller number of neurons (20 instead of 94). Overall CILDS extracts latent variables that more closely match the ground truth simulated latent variables than the other two methods, consistent with Fig. 3. Black circles represent the parameter settings that are fixed across all panels in each row. Comparing to Fig. 3, there are two notable features. First, when the calcium indicator decay is slow, it becomes even more important to deconvolve. Recall that CILDS and deconv-LDS both include deconvolution, whereas LDS does not. CILDS performs similarly whether the calcium indicator is fast (Fig. 3d) or slow (here in panel a). The same is true for deconv-LDS. By contrast, LDS performs worse for slow compared to fast calcium indicator decay because it does not include deconvolution. Second with fewer neurons, the performance of all methods goes down. And as a result, there is a smaller difference in performance between methods (here in panel b). With less statistical power to leverage for separating calcium decay and latent timescale, the three methods show less distinction in performance.

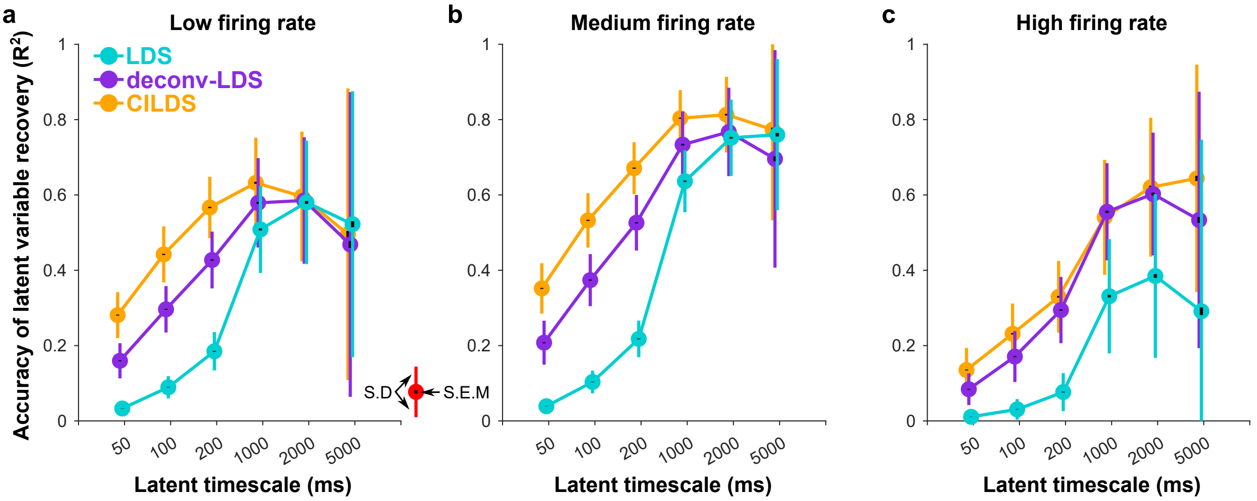

Supplementary Figure 2. Accuracy of latent variable recovery in different simulated firing rate regimes. Here we explored the sensitivity of the three dimensionality reduction approaches to neuron firing rates. In our simulations, we defined firing rates as $\log(1+\exp(Wz_t+\mu))$, where $W \in \mathbb{R}^{q\times p}$ is the loading matrix, $z_t \in \mathbb{R}^{p\times 1}$ is the latent variable at each time point, $\mu \in \mathbb{R}^{q\times 1}$ is a constant offset vector, p is the number of latent variables, and q is the number of neurons. Here the log and exp functions are performed element-by-element as a rectifying nonlinearity. To specifically evaluate the sensitivity that the different dimensionality reduction techniques might have to firing rate, we performed a new analysis in which we ran simulations with three constant values of $\mu \in \{1,10,100\}$, where every element of μ was set to the same value. We label these values as **(a)** low firing rate, **(b)** medium firing rate, and **(c)** high firing rate, respectively. We plot here the R^2 measured between the ground truth latent variables and the extracted latent variables, using the same conventions as in Fig. 3d. As in Fig. 3d, the simulation parameters were set to 94 neurons, a calcium decay corresponding to GCaMP6f, and medium fluorescence noise. For context, in our main simulations (Figs. 3, 4, Supplementary Figs. 1, 3, 4), (Supplementary Table 1) was estimated from electrophysiology recordings and was therefore different for each neuron (14.0 ± 6.8, mean $\pm$ standard deviation), similar to the medium firing rate regime here. We found that CILDS continues to perform better than or equivalently to the other two approaches in accuracy of latent variable recovery regardless of firing rate regime. We also note that there are differences in the overall accuracy of latent variable recovery across firing rate regimes, with the highest accuracy of latent variable recovery found at medium firing rates. When μ is small, the soft rectification represented by the $\log(1+\exp(Wz_t+\mu))$ term tends to squash changes in z_t. As a result, the linear dimensionality reduction methods are less able to recover the underlying latent variables z_t. When μ is large, there would be greater variance in the resulting inhomogeneous Poisson spike trains even though the variance of z_t (which is the "signal" being recovered) remains the same. This makes it more difficult for the dimensionality reduction methods to recover the latent variables.

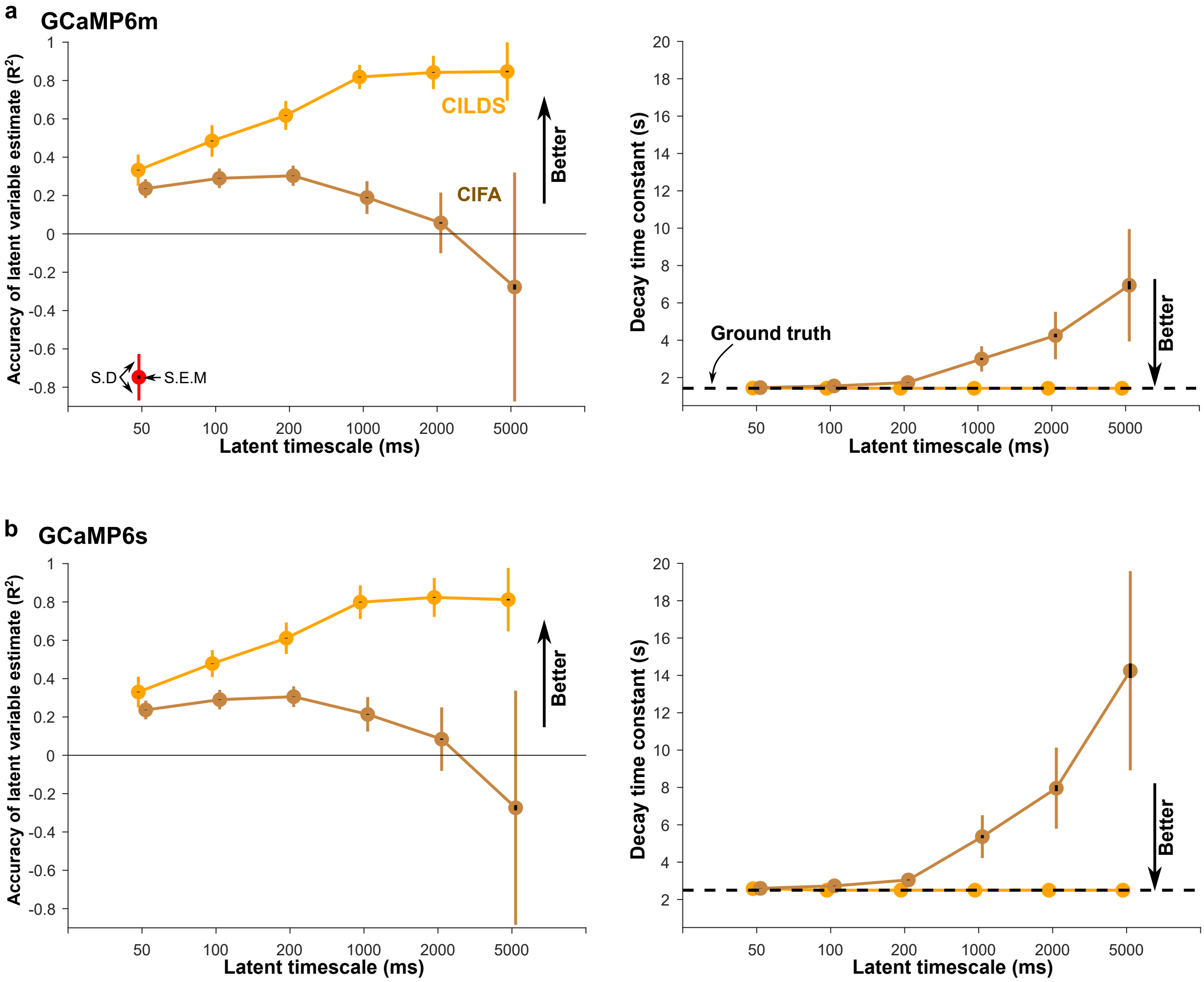

Supplementary Figure 3. Comparison of CILDS and CIFA for different calcium indicators. Same conventions as Fig. 4. Here we compare between CILDS and CIFA for (a) GCaMP6m and (b) GCaMP6s, instead of GCaMP6f (Fig. 4). Consistent with Fig. 4, the overestimation of the calcium indicator decay time constant for CIFA increases as the latent timescales increase. The overestimation also increases when progressing from GCaMP6f (Fig. 4, note different vertical scale) to GCaMP6m (panel a) to GCaMP6s (panel b).

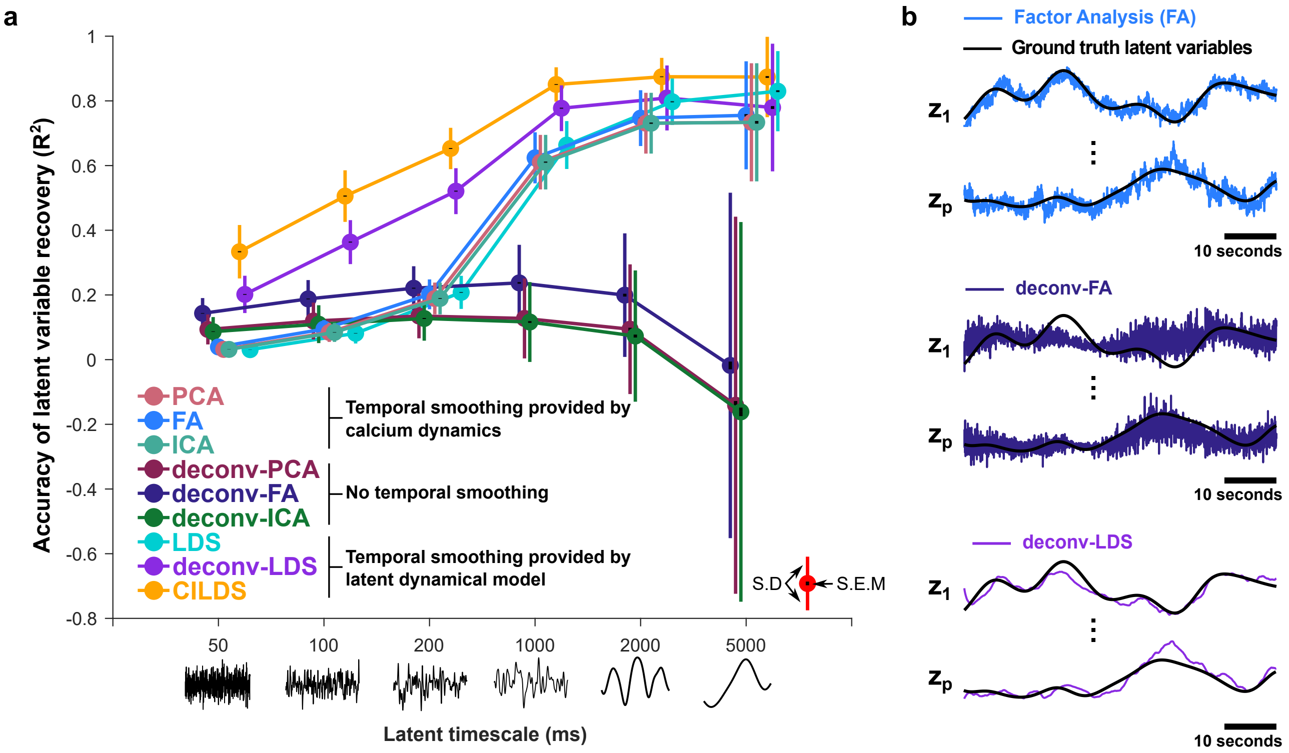
a
b
Accuracy of latent variable recovery (R^2)
1
0.8
0.6
0.4
0.2
0
-0.2
-0.4
-0.6
-0.8
PCA
FA
ICA
deconv-PCA
deconv-FA
deconv-ICA
LDS
deconv-LDS
CILDS
Temporal smoothing provided by calcium dynamics
No temporal smoothing
Temporal smoothing provided by latent dynamical model
S.D
S.E.M
50
100
200
1000
2000
5000
Latent timescale (ms)
Factor Analysis (FA)
Ground truth latent variables
z_1
z_p
10 seconds
deconv-FA
z_1
z_p
10 seconds
deconv-LDS
z_1
z_p
10 seconds

Supplementary Figure 4. Accuracy of latent variable recovery for CILDS compared to standard dimensionality reduction methods. **(a)** We assessed the performance of CILDS relative to standard dimensionality reduction methods: factor analysis (FA), principal components analysis (PCA), and independent components analysis (ICA). Similar to our treatment of LDS, we applied these dimensionality reduction methods using two approaches. In the first approach, we apply the dimensionality reduction method directly to the simulated fluorescence traces. In the second approach, we first deconvolve the fluorescence traces, then apply dimensionality reduction, as in deconv-PCA, deconv-FA, and deconv-ICA. We then assessed the accuracy of latent variable recovery over a range of latent timescales, shown here with the same conventions as Fig. 3d. We found that CILDS outperforms these other dimensionality reduction methods across the range of latent timescales. In addition, when the latent timescales are faster (left side), deconvolving first (deconv-PCA, deconv-FA, and deconv-ICA) aids in better latent variable recovery than applying dimensionality reduction directly to the fluorescence traces (PCA, FA, ICA). However, when the latent timescales are slower (right side), methods which do not deconvolve (PCA, FA, ICA) outperform those which do deconvolve (deconv-PCA, deconv-FA, and deconv-ICA). The reason is that, for the methods which do not deconvolve, the temporal smoothing imposed by the calcium transients shows up in the latent variable estimates. This can be beneficial to latent variable recovery if the latent variables happen to vary as slowly as the calcium transients. The CILDS and deconv-LDS curves shown here are the same as in Fig. 3d. **(b)** To verify this intuition, we show latent variables extracted by three representative methods FA (top, light blue), and deconv-FA (middle, dark blue), deconv-LDS (bottom, purple) compared to the ground truth latent variables (black). In this case, the ground truth latent variables have a timescale of 5000 ms. The latent variables estimated by FA are somewhat smooth due to the calcium transients inherent to the fluorescence traces. By contrast, if we first deconvolve the fluorescence before applying FA (deconv-FA), the estimated latent variables have no temporal smoothing. By including a latent dynamical model (deconv-LDS), we first deconvolve to remove the temporal smoothing imposed by calcium transients. The LDS then applies an appropriate degree of temporal smoothing to the latent variables, as determined by the data. In this simulation, we used a setting of 94 neurons, GCaMP6f, and medium noise. FA and PCA were implemented using custom MATLAB scripts. For ICA, we used MATLAB's built-in `rica` function.

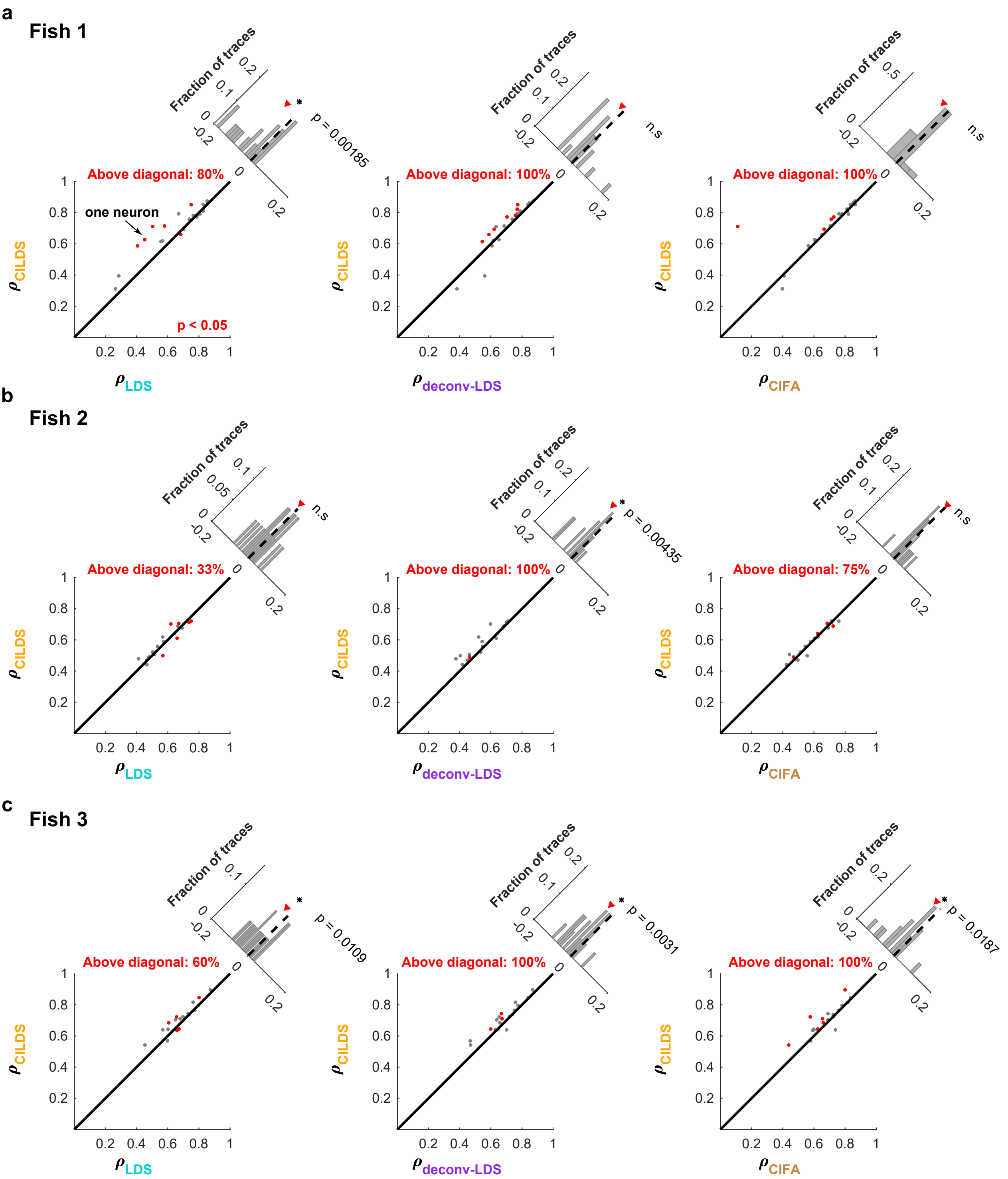

Supplementary Figure 5. Performance comparison on individual larval zebrafish. Same conventions as Fig. 5c-e. **(a)** From left to right, correlations between recorded fluorescence and leave-neuron-out predicted fluorescence, comparing CILDS to LDS, CILDS to deconv-LDS, and CILDS to CIFA for Fish 1. **(b)** Same as (a), but for Fish 2. **(c)** Same as (a), but for Fish 3. These results are consistent with Fig. 5c-e. Indicated is the percentage of significant neurons (i.e., percentage of red points) above the diagonal. Note that the threshold used for the t-test means that we might expect 5% of the neurons to appear significant even if the effect is not real.

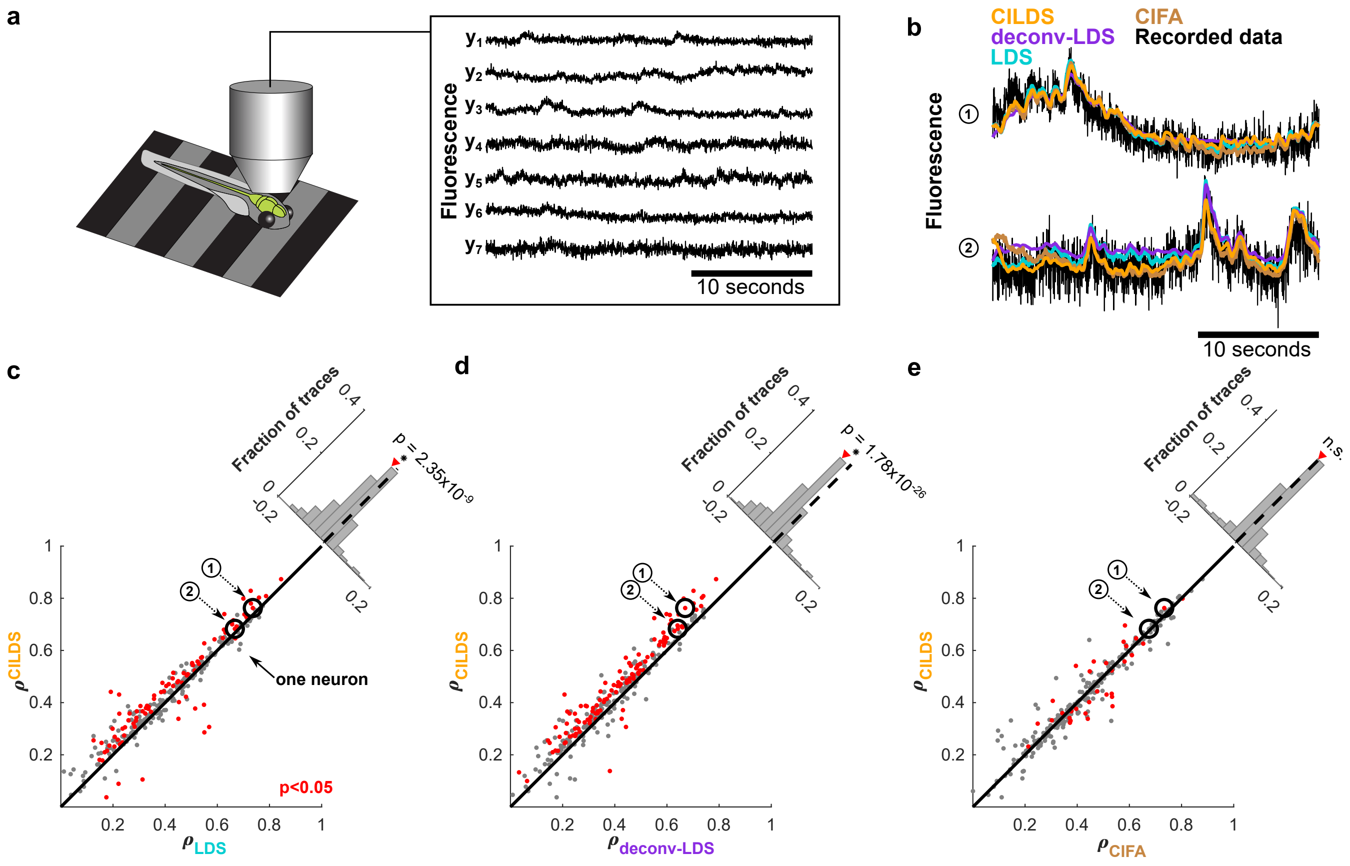
a
y1
y2
y3
y4
y5
y6
y7
Fluorescence
10 seconds
b
CILDS
CIFA
deconv-LDS
Recorded data
LDS
Fluorescence
10 seconds
c
Fraction of traces
p = 2.35x10-9
one neuron
p<0.05
ρCILDS
ρLDS
d
Fraction of traces
p = 1.78x10-26
ρCILDS
ρdeconv-LDS
e
Fraction of traces
n.s.
ρCILDS
ρCIFA

Supplementary Figure 6. Performance comparison on larval zebrafish experiment with recordings from hundreds of neurons spanning multiple brain areas. Neurons were imaged from a single plane of a larval zebrafish brain while it engaged in “fictive swimming". Calcium imaging was performed using light-sheet microscopy at 66 Hz in a single plane on *Tg(elavl3:GCaMP6f)*^{jf1} fish expressing GCaMP6f in the cytosol of neurons[63]. The segmentation algorithm for cell identification was a modified version of a previously-proposed method with multiple stages that goes from spatiotemporal denoising to demixing[67]. In the experiment, the neurons were imaged in two conditions, randomly interleaved: (1) fictive swimming in closed loop with backward visual flow to mimic “successful” swimming (95% of swims), and (2) swimming in open loop without visual feedback to mimic “failed” swims (5% of swims). The imaging session was divided evenly into 10 trials, each 76.4 seconds long. From the single plane imaging, we computed leave-neuron-out predictions using 10-fold cross-validation with 300 of the imaged neurons selected at random from the entire plane, spanning multiple brain areas (35% from forebrain, 30% from midbrain, 35% from hindbrain). Same conventions as Fig. 5. **(a)** Representative fluorescence traces from seven of the imaged neurons. **(b)** Example recorded fluorescence traces (black) and leave-neuron-out predicted fluorescence using CILDS (orange), deconv-LDS (purple), LDS (cyan), and CIFA (brown). **(c-e)** Correlation between the recorded fluorescence and the leave-neuron-out predicted fluorescence for CILDS versus each of the other methods. Each point represents one neuron, where the correlation is computed for each trial (76.4 seconds long) then averaged across all 10 trials. Diagonal histograms show the paired difference in performance between CILDS and one of the other methods, as indicated. The correlation is higher for CILDS than (c) LDS ($p < 1 \times 10^{-8}$, n=300 neurons, paired two-tailed t-test across the population of neurons), (d) deconv-LDS ($p < 1 \times 10^{-25}$, $n = 300$ neurons), and on par with (e) CIFA (n.s., $n = 300$ neurons). This is consistent with the other zebrafish experiments in this paper when we analyze each zebrafish individually (Supplementary Fig. 5). Note that the histograms are zoomed-in for visual clarity, and therefore the ends of the histograms are not shown. The numbered points (black circles) correspond to the examples shown in panel b. Red points indicate a statistically significant difference per neuron between CILDS and the other method being compared using a paired two-tailed t-test across trials ($p < 0.05$, see Methods). Note that the threshold used for the t-test means that we might expect 5% of the neurons to appear significant even if the effect is not real. Here again we found that CILDS outperforms deconv-LDS (87% of red points above the diagonal) and LDS (97% of red points above the diagonal), and performs similarly to CIFA (47% of red points above the diagonal). To contextualize these findings, we found in simulation that even when CILDS is substantially more accurate than CIFA at estimating the latent variables, the two methods can perform similarly in terms of leave-neuron-out prediction (Supplementary Fig. 12). We have evidence that this may also be the case for the calcium imaging recordings. The telltale sign is that, from these zebrafish recordings, CIFA estimates a calcium decay constant of 9 seconds (median across 300 neurons), which is far greater than what is expected of GCaMP6f in larval zebrafish. By contrast, CILDS estimates a calcium decay constant of 2 seconds (median across 300 neurons), which is consistent with what would be expected of GCaMP6f in larval zebrafish[40].

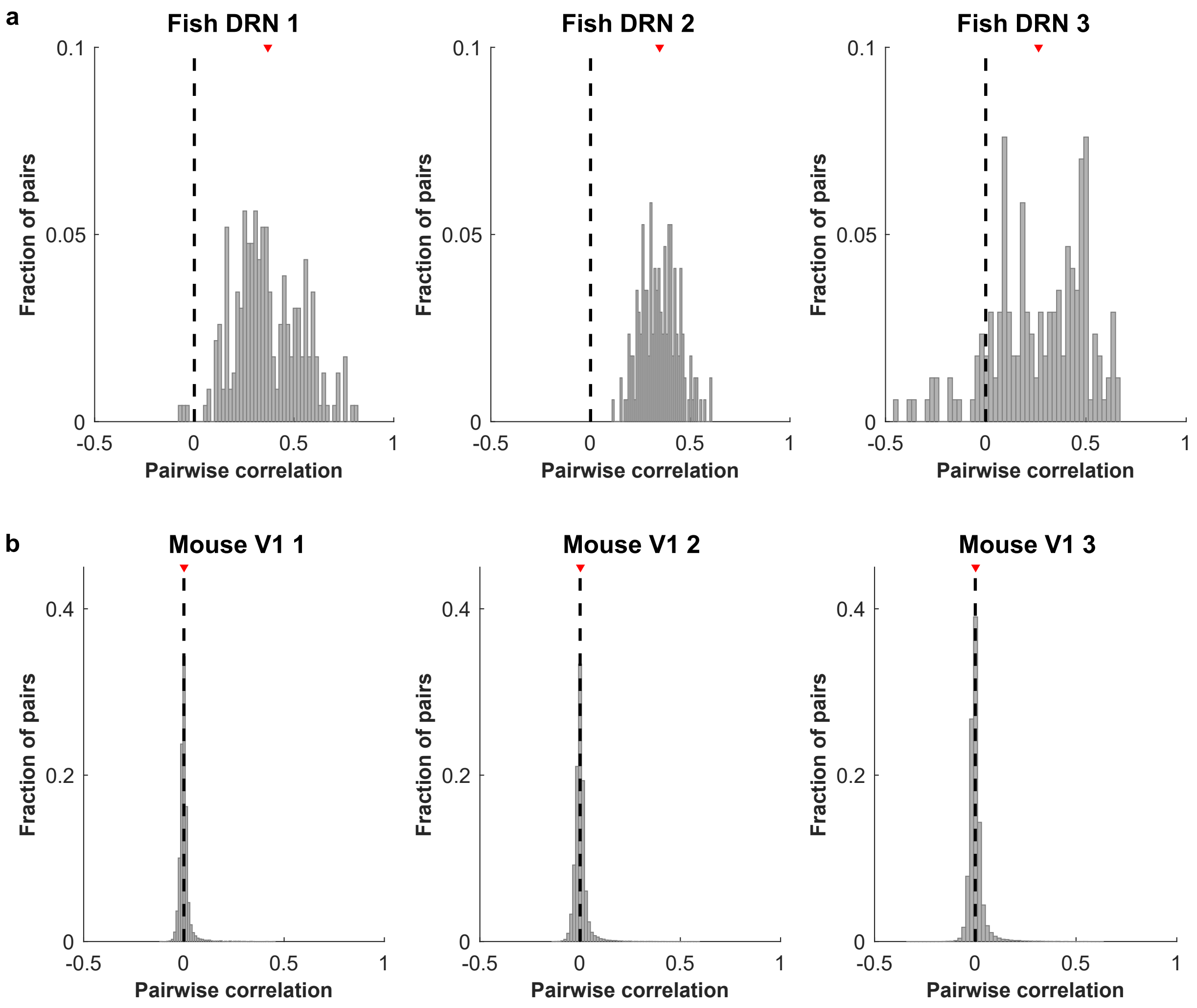

Supplementary Figure 7. Activity correlations between pairs of neurons for larval zebrafish DRN and mouse V1. Pairwise correlations between neurons across trials for the (a) larval zebrafish DRN neurons (0.50 ± 0.07, mean $\pm$ standard deviation) and (b) mouse V1 neurons (0.0015 ± 0.0005, mean $\pm$ standard deviation). The mean is indicated by red triangles. Despite the small mean pairwise correlations in the mouse V1 data, there exists shared variability within the neural population[44,45], as indicated by the ability to reconstruct fluorescence traces in the leave-one-out neuron analysis of Fig. 6c-e. Consistent with the difference in pairwise correlations, the absolute level of predictability is lower for mouse V1 than for fish DRN (Fig. 5, 6).

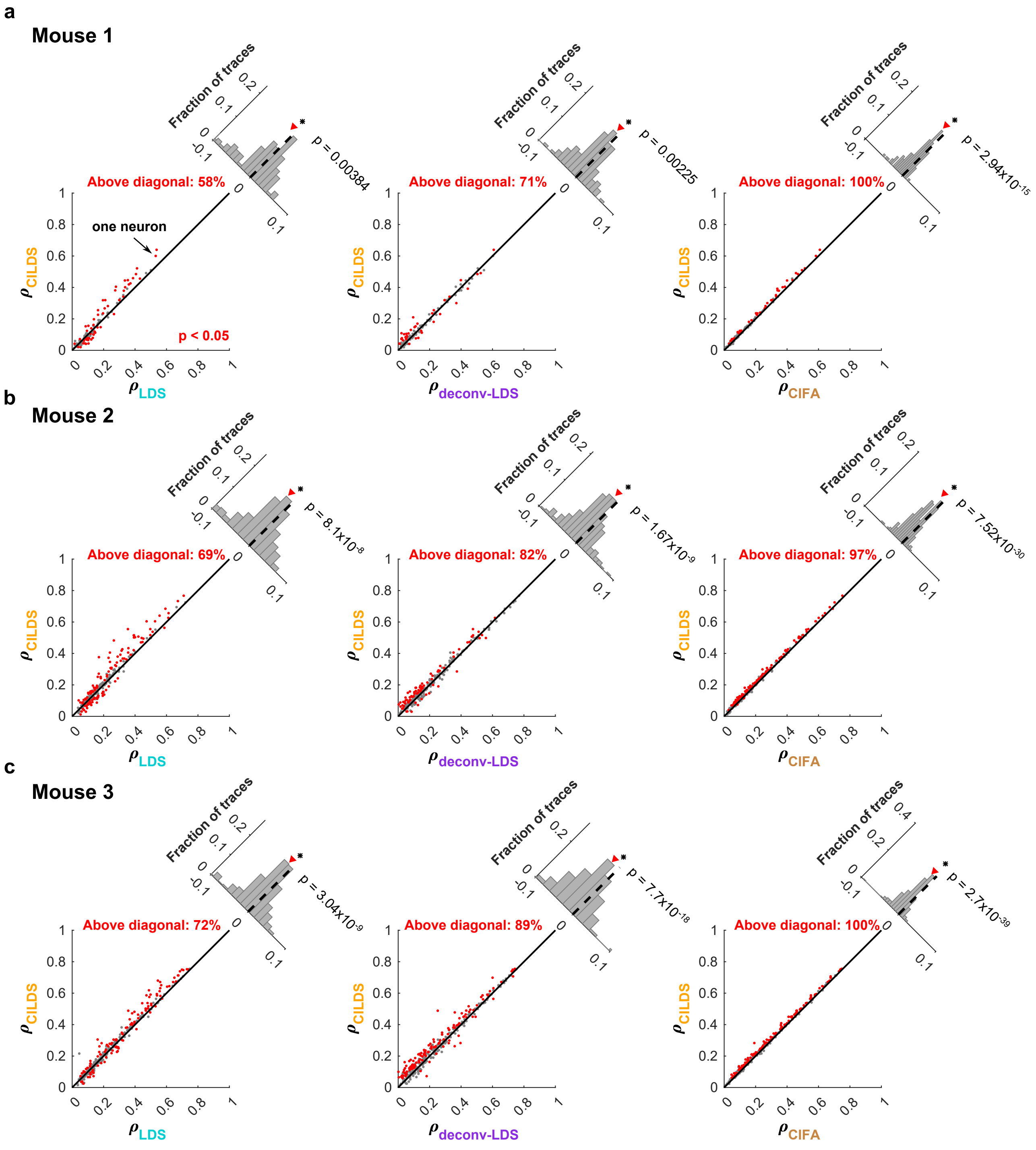

Supplementary Figure 8. Performance comparison on individual mice. Same conventions as Fig. 6c-e. **(a)** From left to right, correlations between recorded fluorescence and leave-neuron-out predicted fluorescence, comparing CILDS to LDS, CILDS to deconv-LDS, and CILDS to CIFA for Mouse 1. **(b)** Same as (a), but for Mouse 2. **(c)** Same as (a), but for Mouse 3. These results are consistent with Fig. 6c-e, for each mouse individually. Indicated is the percentage of significant neurons (i.e., percentage of red points) above the diagonal. Note that the threshold used for the t-test means that we might expect 5% of the neurons to appear significant even if the effect is not real.

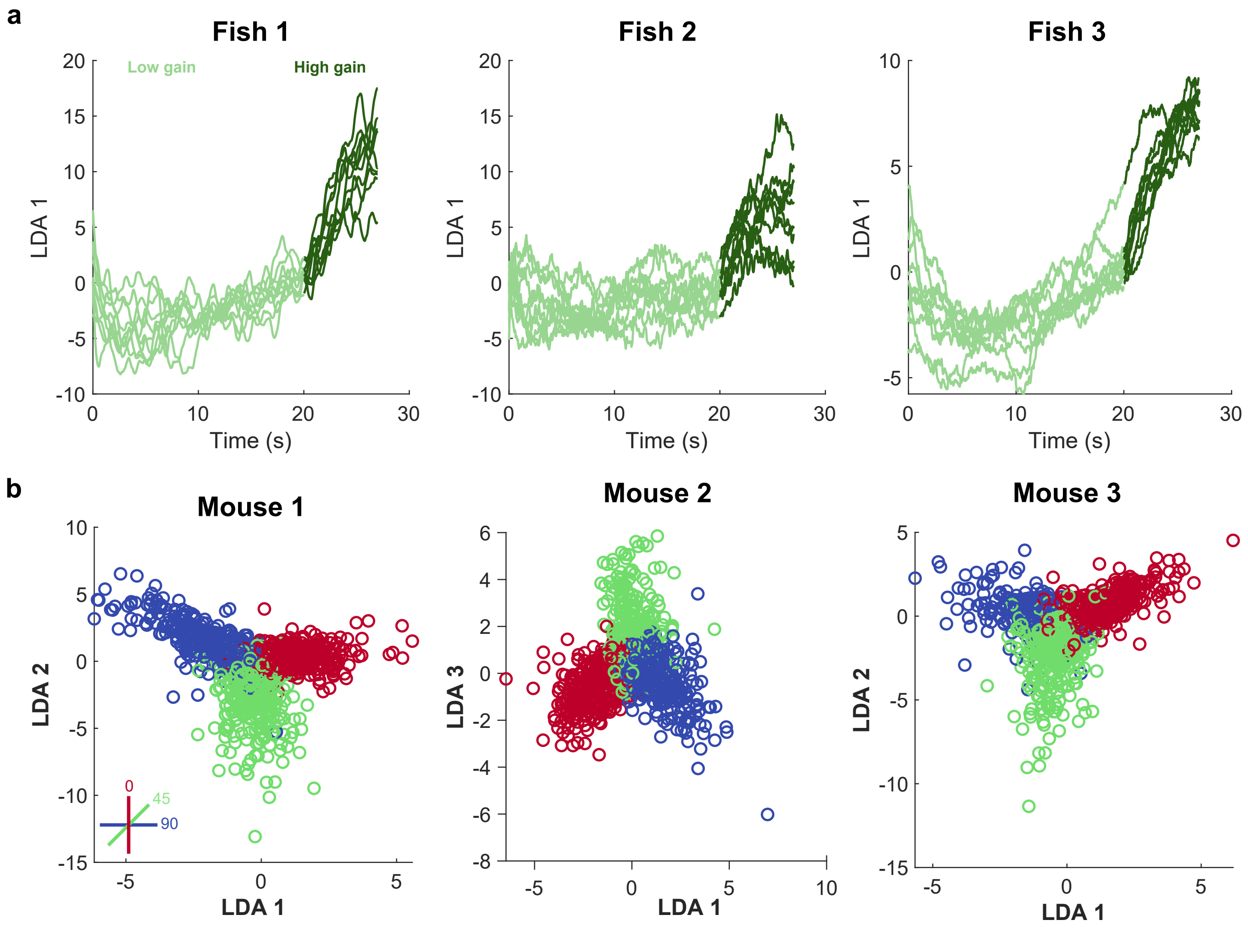
a
Fish 1
Fish 2
Fish 3
Low gain
High gain
LDA 1
Time (s)
b
Mouse 1
Mouse 2
Mouse 3
LDA 1
LDA 2
LDA 3
0
45
90

Supplementary Figure 9. Exploratory data analyses using CILDS. Here we visualize how the latent variables extracted by CILDS vary with the behavioral context or sensory stimuli. We first applied CILDS to the fluorescence traces of each animal to extract low-dimensional latent variables. For visualization, we then applied linear discriminant analysis (LDA) to the latent variables extracted via CILDS. This allows us to find projections that best separate neural activity with respect to different experimental variables. **(a)** For the larval zebrafish DRN experiments, the variable being manipulated was the gain between the movement of the visual stimulus and the fish's motor response[40]. The fish experienced both a low gain setting to increase locomotor drive and a high gain setting to attenuate their locomotor drive (see Methods). To demonstrate how the gain affected the latent variables extracted by CILDS, we applied LDA to identify the axis that best separates low gain (light green) and high gain (dark green). We see that there is a clear distinction in the latent activity from the low-gain and high-gain conditions, indicating that CILDS is recovering experimentally-relevant modulations in neural activity. For this analysis, we set the number of dimensions extracted by CILDS to be the mean of the optimal latent dimensionality across outer folds for each fish (between 9 and 17, depending on fish) from nested cross-validation (see Methods). **(b)** For the mouse V1 experiments, neural activity was driven by different presented visual gratings (see Methods and Jeon et al. 2018[41] for more details). To visualize the latent variables extracted by CILDS, we applied LDA to the latent variables to find the two-dimensional plane that best separated the 12 different grating orientations (where recordings were combined across the different spatial frequencies). Here we show three of these orientations, 0 (red), 45 (green), and 90 (blue). We observed distinct orientation-specific clusters, indicating a reliable modulation of the CILDS-extracted latent variables with the visual stimulus. For this analysis, we set the number of dimensions extracted by CILDS to be 50.

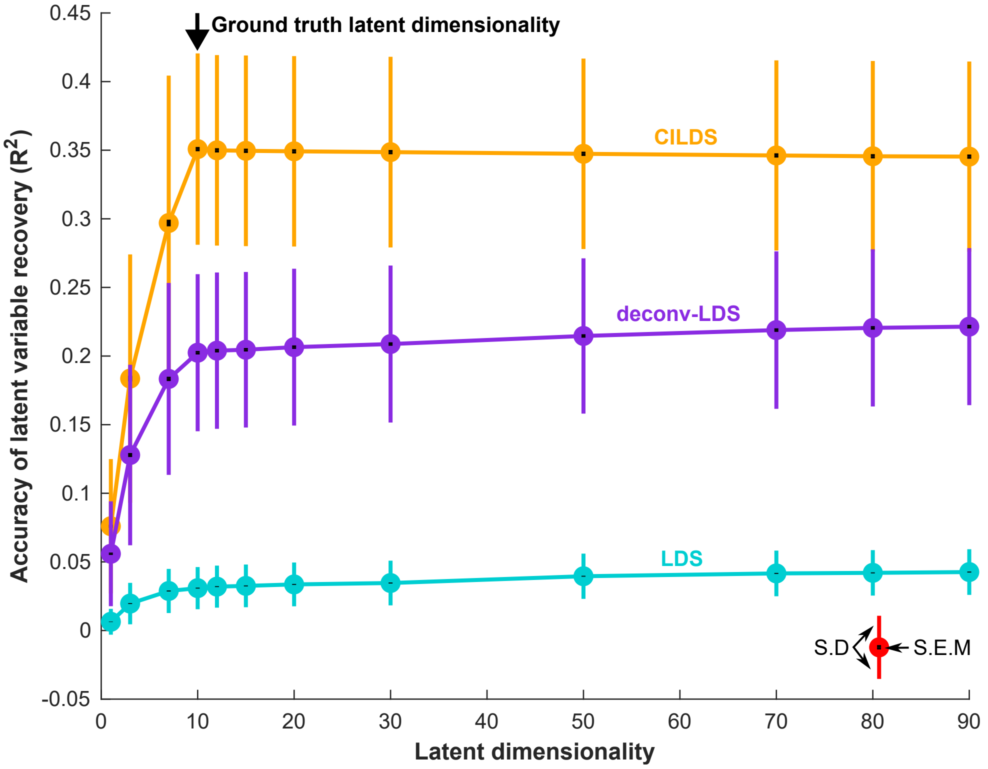

Supplementary Figure 10. Accuracy of latent variable recovery while varying the ground truth latent dimensionality. To understand how the accuracy of latent variable recovery is influenced by the choice of the number of latent variables extracted by each method (i.e., latent dimensionality), we systematically varied the latent dimensionality and evaluated the R^2 between the ground truth and estimated latent variables using CILDS (orange), deconv-LDS (purple), and LDS (cyan). In this simulation, the ground truth latent dimensionality was fixed at 10, as in Fig. 3. If we extract too few latent variables (less than 10), the performance of all methods degrades quickly. If we instead extract too many latent variables (greater than 10), the performance tends to be stable. Note that when fitting more latent variables than the ground truth latent dimensionality, our transformation matrix W has dimensions $a \times p$, where a is the selected latent dimensionality and p is the ground truth latent dimensionality (see Evaluation of dimensionality reduction methods in Methods). These simulations are in a data rich regime (240,000 data points). Thus we tend to observe stable performance rather than overfitting when the number of extracted latent variables is large.

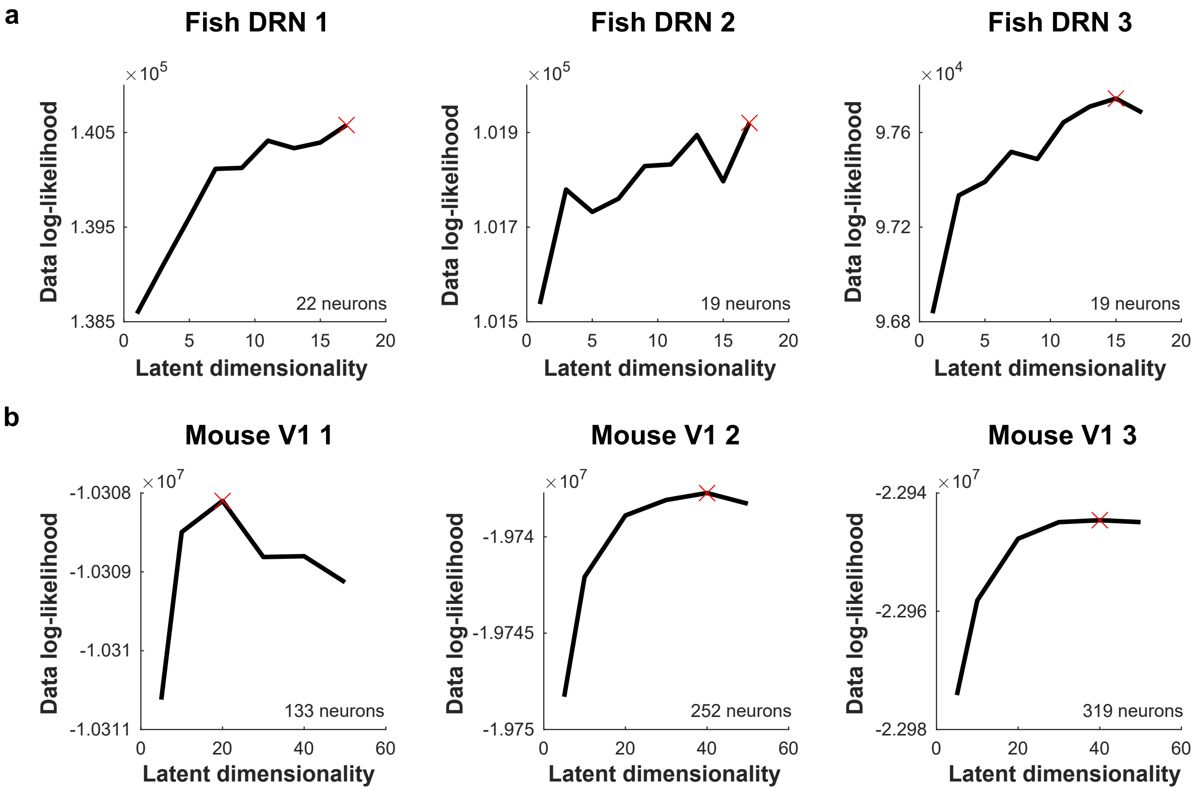

Supplementary Figure 11. Model selection to assess latent dimensionality. A common way of selecting the latent dimensionality for dimensionality reduction is to vary the latent dimensionality and compute the data likelihood ($P(\{y\})$, defined based on equations (6) - (10)), in a cross-validated manner (see Methods for cross-validation procedure). Here we show how the cross-validated data log-likelihood varies with latent dimensionality for CILDS in **(a)** the larval zebrafish DRN and **(b)** mouse V1, with the peak data log-likelihood marked in red.

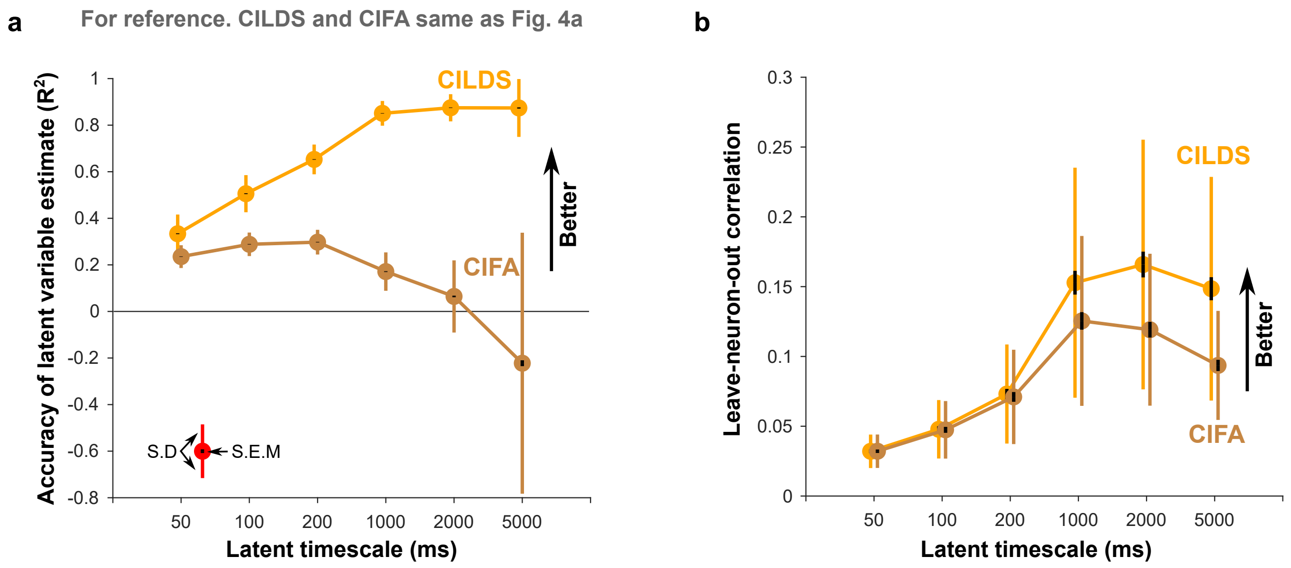

Supplementary Figure 12. Relationship between the two performance metrics: accuracy of latent variable recovery and leave-neuron-out prediction. In simulation, CILDS tends to substantially outperform CIFA at latent variable recovery (Fig. 4a). For experimental data, we do not have the ground truth latent variables, and so we use a leave-neuron-out prediction of fluorescence. Across the zebrafish (Fig. 5) and mice experiments (Fig. 6), we found that the difference in leave-neuron-out prediction performance between CILDS and CIFA tends to be small, in contrast to the simulation results. We wondered to what extent this could be due to the use of two different performance metrics: accuracy of latent variable recovery for simulations and leave-neuron-out prediction for experimental data. To better understand these differences, we computed the leave-neuron-out prediction performance in the simulations. **(a)** Reproduced from Fig. 4a, shown here for reference. **(b)** Leave-neuron-out prediction performance across latent timescales for CILDS (orange) and CIFA (brown) for the same simulated data as in panel a. See Methods for details on the leave-neuron-out prediction procedure. We found that the accuracy of latent variable recovery between CILDS and CIFA can be substantial (panel a) even though the difference in leave-neuron-out performance is small (panel b). To understand this, recall that CIFA erroneously attributes the slower varying fluorescence to a slower calcium decay, rather than to a longer latent timescale (Fig. 4b). This error leads to less accurate latent variable recovery. However, when reconstructing fluorescence, CIFA can utilize an overly-slow calcium decay to compensate for misestimated latent variables, allowing it to perform similarly to CILDS in the leave-neuron-out correlation metric.

**Parameters**	**Variables**
q: No. of neurons	20, 50, 94
p: No. of latent variables	10
N: No. of trials	200
T: Length of trial	60 seconds
W: Loading matrix	Estimated from electrophysiological recordings using FA[6]
μ: Mean firing rate	Estimated from electrophysiological recordings data using FA[6]
B: Non-negative diagonal matrix	I
$\boldsymbol{b}$: Baseline fluorescence	$\mathbf{0}$
τ: Latent timescale	50, 100, 200, 1000, 5000 ms
Γ: GCaMP6 decay	f: $(0.9985)I$, m: $(0.9993)I$, s: $(0.9996)I$
R: Fluorescence noise	low:$(0.15)I$, medium: $(1.5)I$, high: $(15)I$

Supplementary Table 1. Parameter values used for simulations. Parameters within the simulation framework that were varied, and the range of parameter values tested. We chose parameter values to mimic those in real data.

Supplemental Information

Practical considerations

Approximating a long Gaussian process

To carry out computations using a GP, one would need to represent a covariance matrix of size $T \times T$ in memory, where T =6,000,000 in our simulations. For such values of T, the memory requirement can exceed the memory capacity of the computer. To overcome this, we employed two strategies in tandem. First, we generated the GPs one segment at a time, rather than all T time points at once. For example, to generate a GP with 6,000,000 time points, we can first generate a GP with 5,000 time points according to equations (12) and (13). Then, we can generate a GP for the next 5,000 time points conditioned on the first 5,000 time points using Gaussian conditioning

$$\begin{bmatrix} \boldsymbol{x} \\ \boldsymbol{y} \end{bmatrix} \sim \mathcal{N}\left(\begin{bmatrix} \mathbf{0} \\ \mathbf{0} \end{bmatrix}, \begin{bmatrix} A & C \\ C^T & B \end{bmatrix} \right) \tag{19}$$

$$\boldsymbol{x}|\boldsymbol{y} \sim \mathcal{N}(CB^{-1}\boldsymbol{y}, A - CB^{-1}C^T) \tag{20}$$

where $A \in \mathbb{R}^{5000\times5000}$ and $B \in \mathbb{R}^{5000\times5000}$ are the covariances of the second and first half of the GP, respectively. $C \in \mathbb{R}^{5000\times5000}$ is the covariance between the first and second half of the GP. We can continue this procedure, where for each new segment we condition on all of the segments that have been generated thus far. Statistically this procedure is equivalent to if we had generated the entire GP time series at once.

As we continue this procedure, the number of time points being conditioned on will grow and the matrices B and C in equations (19) and (20) can exceed the memory capacity of the computer. We thus employ a second strategy that leverages the fact that, according to the squared exponential covariance (13), two time points covary highly when they are close in time and almost independent when they are far apart in time. Thus, we make the approximation that in equations (19) and (20), we condition only on the most recent 5,000 time points.

Computational running time

The following are representative running times to fit the different dimensionality reduction models. These running times are based on single threads run on Matlab (2019a) using Intel(R) Xeon(R) CPU processors (Gold 6230, 2.1 GHz) with 250 GB of RAM. First, consider one cross-validation fold of the zebrafish recordings with 4 trials, 22 neurons, 10 latent variables, and 1950 time points per trial. For each EM iteration, CILDS takes on average approximately 0.9 s, LDS (as well as the second-stage of deconv-LDS) takes 0.4 s. Second, consider one cross-validation fold of the mouse recordings with 12 trials, 319 neurons, 30 latent variables, and 3049 time points per trial. For each EM iteration, CILDS takes on average approximately 110 s, and LDS (as well as the second-stage of deconv-LDS) takes 10 s. For all methods, the most expensive computations are the matrix

inversions in the expectation step of the EM algorithm. There is a $p \times p$ matrix inversion at each time point for LDS (and deconv-LDS), and a $(p+q) \times (p+q)$ matrix inversion at each time point for CILDS, where p is the latent dimensionality and q is the number of neurons.

EM algorithm for CILDS

The CILDS model is defined by equations (6) - (10). Only the fluorescence values $\boldsymbol{y}_t$ are observed, whereas the calcium concentrations $\boldsymbol{c}_t$ and latent variables $\boldsymbol{z}_t$ are not observed.

The goal of the EM algorithm is to maximise the probability of the observed fluorescence traces $P(\{\boldsymbol{y}\})$ with respect to the model parameters $\theta := \{D, P, \boldsymbol{h}_2, G_2, \Gamma, A, \boldsymbol{b}, Q, B, R, \boldsymbol{\mu}_1, V_1\}$, where $\{\boldsymbol{y}\}$ is shorthand for $\boldsymbol{y}_1, ..., \boldsymbol{y}_T$. To perform this maximization, we iteratively perform an expectation step (E-step), then a maximization step (M-step), as detailed below.

1 Expectation Step

The goal of the E-step is to compute the posterior distribution $s := P(\{\boldsymbol{c}\}, \{\boldsymbol{z}\} | \{\boldsymbol{y}\})$. Using this posterior distribution, we can compute the following expectations

$$E_s\left[\boldsymbol{c}_{t-1}^{(i)}\right], E_s\left[\boldsymbol{c}_t^{(i)}\right], E_s\left[\boldsymbol{c}_t^{(i)}\boldsymbol{c}_t^{(i)\prime}\right], E_s\left[\boldsymbol{c}_t^{(i)}\boldsymbol{c}_{t-1}^{(i)\prime}\right], E_s\left[\boldsymbol{c}_t^{(i)}\boldsymbol{z}_t^{(i)\prime}\right]$$

$$E_s\left[\boldsymbol{c}_{t-1}^{(i)}\boldsymbol{z}_t^{(i)\prime}\right], E_s\left[\boldsymbol{z}_t^{(i)}\boldsymbol{z}_t^{(i)\prime}\right], E_s\left[\boldsymbol{z}_t^{(i)}\boldsymbol{z}_{t-1}^{(i)\prime}\right], E_s\left[\boldsymbol{z}_t^{(i)}\right], E_s\left[\boldsymbol{z}_{t-1}^{(i)}\right]$$

which are needed in the M-step. We start by rewriting equations (7) and (9) in block matrix notation

$$\begin{bmatrix} \boldsymbol{c}_t \\ \boldsymbol{z}_{t+1} \end{bmatrix} = \begin{bmatrix} \Gamma & A \\ 0 & D \end{bmatrix} \begin{bmatrix} \boldsymbol{c}_{t-1} \\ \boldsymbol{z}_t \end{bmatrix} + \begin{bmatrix} \boldsymbol{b} \\ \boldsymbol{0} \end{bmatrix} + \begin{bmatrix} \boldsymbol{w}_t \\ \boldsymbol{v}_{t+1} \end{bmatrix}.$$

The observation model (6) can be written as

$$\boldsymbol{y}_t = \Phi \begin{bmatrix} \boldsymbol{c}_t \\ \boldsymbol{z}_{t+1} \end{bmatrix} + \boldsymbol{\varepsilon}_t, \quad \text{where } \Phi := \begin{bmatrix} B & 0 \end{bmatrix}.$$

We define $\boldsymbol{l}_t := \begin{bmatrix} \boldsymbol{c}_t \\ \boldsymbol{z}_{t+1} \end{bmatrix}, \Lambda := \begin{bmatrix} \Gamma & A \\ 0 & D \end{bmatrix}, \boldsymbol{m} := \begin{bmatrix} \boldsymbol{b} \\ \boldsymbol{0} \end{bmatrix}, \boldsymbol{n}_t := \begin{bmatrix} \boldsymbol{w}_t \\ \boldsymbol{v}_{t+1} \end{bmatrix}, S := \begin{bmatrix} Q & 0 \\ 0 & P \end{bmatrix}, \boldsymbol{\eta}_1 := \begin{bmatrix} \boldsymbol{\mu}_1 \\ \boldsymbol{h}_2 \end{bmatrix}, \Sigma_1 := \begin{bmatrix} V_1 & 0 \\ 0 & G_2 \end{bmatrix}.$

The CILDS model can thus be written in block matrix notation as

$$\boldsymbol{y}_t = \Phi \boldsymbol{l}_t + \boldsymbol{\varepsilon}_t \tag{21}$$

$$\boldsymbol{l}_t = \Lambda \boldsymbol{l}_{t-1} + \boldsymbol{m} + \boldsymbol{n}_t \tag{22}$$

where $\boldsymbol{l}_1 \sim \mathbb{N}(\boldsymbol{\eta}_1, \Sigma_1)$, $\boldsymbol{n}_t \sim \mathbb{N}(\boldsymbol{0}, S)$, and $t = 1, \ldots, T$. In other words, CILDS can be written as an LDS whose parameters are constrained in a specific way. We seek to compute $P(\boldsymbol{l}_t, \boldsymbol{l}_{t-1} | \{\boldsymbol{y}\}_1^T)$ for $t = 2, \ldots, T$. This distribution is Gaussian, and thus it is sufficient to find its mean and covariance. We denote $E\left[\boldsymbol{l}_t | \{\boldsymbol{y}\}_1^{\tau}\right]$ by $\boldsymbol{l}_t^{\tau}$ and $Var\left[\boldsymbol{l}_t | \{\boldsymbol{y}\}_1^{\tau}\right]$ by V_t^{τ}. For brevity, we only show the results of the derivations below.

1.1 Forward Recursions

To obtain $\boldsymbol{l}_t^t$ and V_t^t, we recursively compute the following equations from $t = 1$ to $t = T$

$$
\begin{aligned}
\boldsymbol{l}_t^{t-1} &= \Lambda \boldsymbol{l}_{t-1}^{t-1} + \boldsymbol{m} \\
V_t^{t-1} &= \Lambda V_{t-1}^{t-1} \Lambda' + S \\
K_t &= V_t^{t-1} \Phi' \left(R + \Phi V_t^{t-1} \Phi'\right)^{-1} \\
\boldsymbol{l}_t^t &= \boldsymbol{l}_t^{t-1} + K_t \left(\boldsymbol{y}_t - \Phi \boldsymbol{l}_t^{t-1}\right) \\
V_t^t &= V_t^{t-1} - K_t \Phi V_t^{t-1}.
\end{aligned}
$$

The recursions are initialized with $\boldsymbol{l}_1^0 = \eta_1$, $V_1^0 = \Sigma_1$.

1.2 Backward Recursions

To obtain $\boldsymbol{l}_t^T$ and V_t^T, we recursively compute the following equations from $t = T$ to $t = 2$. We also compute the covariance of the joint posterior distribution $P(\boldsymbol{l}_t, \boldsymbol{l}_{t-1} | \{\boldsymbol{y}\}_1^T)$, denoted as $V_{t,t-1}^T$

$$
\begin{aligned}
J_{t-1} &= V_{t-1}^{t-1} \Lambda' \left(V_t^{t-1}\right)^{-1} \\
\boldsymbol{l}_{t-1}^T &= \boldsymbol{l}_{t-1}^{t-1} + J_{t-1} \left(\boldsymbol{l}_t^T - \Lambda \boldsymbol{l}_{t-1}^{t-1} - \boldsymbol{m}\right) \\
V_{t-1}^T &= V_{t-1}^{t-1} + J_{t-1} \left(V_t^T - V_t^{t-1}\right) J_{t-1}' \\
V_{t,t-1}^T &= V_t^T J_{t-1}'.
\end{aligned}
$$

2 Maximization Step

In the M-step, we seek to maximize the expected log joint distribution

$$
\mathscr{Q} := E_s \left[\log P\left(\{\boldsymbol{y}\}^N, \{\boldsymbol{c}\}^N, \{\boldsymbol{z}\}^N\right)\right]
$$

with respect to the model parameters, where $s := P(\{\boldsymbol{c}\}^N, \{\boldsymbol{z}\}^N | \{\boldsymbol{y}\}^N; \theta)$ and $\{\}^N$ represents all T time points across all N trials. The joint distribution for one trial can be factorized as

$$
\begin{aligned}
P(\{\boldsymbol{y}\},\{\boldsymbol{c}\},\{\boldsymbol{z}\}) &= P(\{\boldsymbol{y}\}\,|\,\{\boldsymbol{c}\},\{\boldsymbol{z}\})P(\{\boldsymbol{c}\},\{\boldsymbol{z}\}) \\
&= P(\{\boldsymbol{y}\}\,|\,\{\boldsymbol{c}\})P(\{\boldsymbol{c}\}\,|\,\{\boldsymbol{z}\})P(\{\boldsymbol{z}\}) \\
&= \prod_{t=1}^{T} P(\boldsymbol{y}_t|\boldsymbol{c}_t)\prod_{t=2}^{T} P(\boldsymbol{c}_t|\boldsymbol{c}_{t-1},\boldsymbol{z}_t)\prod_{t=3}^{T} P(\boldsymbol{z}_t|\boldsymbol{z}_{t-1})P(\boldsymbol{c}_1)P(\boldsymbol{z}_2)
\end{aligned} \tag{23}
$$

where these distributions are defined in equations (6) - (10).

$$
\begin{aligned}
\mathscr{Q} &= E_s\left[\log\prod_{i=1}^{N} P\left(\{\boldsymbol{y}\}^{(i)},\{\boldsymbol{c}\}^{(i)},\{\boldsymbol{z}\}^{(i)}\right)\right] \\
&= E_s\left[\sum_{i=1}^{N}\left(-\frac{1}{2}\sum_{t=1}^{T}\left[\boldsymbol{y}_t^{(i)}-B\boldsymbol{c}_t^{(i)}\right]' R^{-1}\left[\boldsymbol{y}_t^{(i)}-B\boldsymbol{c}_t^{(i)}\right]-\frac{T}{2}\log|R|\right.\right. \\
&-\frac{1}{2}\sum_{t=2}^{T}\left[\boldsymbol{c}_t^{(i)}-\Gamma\boldsymbol{c}_{t-1}^{(i)}-A\boldsymbol{z}_t^{(i)}-\boldsymbol{b}\right]' Q^{-1}\left[\boldsymbol{c}_t^{(i)}-\Gamma\boldsymbol{c}_{t-1}^{(i)}-A\boldsymbol{z}_t^{(i)}-\boldsymbol{b}\right]-\frac{T-1}{2}\log|Q| \\
&-\frac{1}{2}\sum_{t=3}^{T}\left[\boldsymbol{z}_t^{(i)}-D\boldsymbol{z}_{t-1}^{(i)}\right]' P^{-1}\left[\boldsymbol{z}_t^{(i)}-D\boldsymbol{z}_{t-1}^{(i)}\right]-\frac{T-2}{2}\log|P| \\
&\left.\left.-\frac{1}{2}\left[(\boldsymbol{z}_2^{(i)}-\boldsymbol{h}_2)'G^{-1}(\boldsymbol{z}_2^{(i)}-\boldsymbol{h}_2)\right]-\frac{1}{2}\log|G|-\frac{1}{2}\left[\boldsymbol{c}_1^{(i)}-\boldsymbol{\mu}_1\right]' V_1^{-1}\left[\boldsymbol{c}_1^{(i)}-\boldsymbol{\mu}_1\right]-\frac{1}{2}\log|V_1|-\frac{T(2q+p)}{2}\log 2\pi\right)\right].
\end{aligned}
$$

To maximize $\mathscr{Q}$ with respect to the model parameters θ, we compute the following partial derivatives

$$
\frac{\partial\mathscr{Q}}{\partial B},\frac{\partial\mathscr{Q}}{\partial R^{-1}},\frac{\partial\mathscr{Q}}{\partial\Gamma},\frac{\partial\mathscr{Q}}{\partial A},\frac{\partial\mathscr{Q}}{\partial\boldsymbol{b}},\frac{\partial\mathscr{Q}}{\partial Q^{-1}},\frac{\partial\mathscr{Q}}{\partial D},\frac{\partial\mathscr{Q}}{\partial P^{-1}},\frac{\partial\mathscr{Q}}{\partial\boldsymbol{\mu}_1},\frac{\partial\mathscr{Q}}{\partial V_1^{-1}},\frac{\partial\mathscr{Q}}{\partial\boldsymbol{h}_2},\frac{\partial\mathscr{Q}}{\partial G_2^{-1}}
$$

and set them to zero to solve for the parameters. Doing so results in the following M-step parameter updates, all of which can be expressed in closed form.

$$
B^{new} = \left(\sum_{i=1}^{N}\sum_{t=1}^{T} diag\{\boldsymbol{y}_t^{(i)}E_s\left[\boldsymbol{c}_t^{(i)'}\right]\}\right)\left(\sum_{i=1}^{N}\sum_{t=1}^{T} diag\{E_s\left[\boldsymbol{c}_t^{(i)}\boldsymbol{c}_t^{(i)'}\right]\}\right)^{-1}
$$

$$
R^{new} = \frac{1}{NT}\sum_{i=1}^{N}\sum_{t=1}^{T}\left(diag\{\boldsymbol{y}_t^{(i)}\boldsymbol{y}_t^{(i)'}\}-2diag\{BE_s\left[\boldsymbol{c}_t^{(i)}\right]\boldsymbol{y}_t^{(i)'}\}+diag\{BE_s\left[\boldsymbol{c}_t^{(i)}\boldsymbol{c}_t^{(i)'}\right]B'\}\right)
$$

$$
\Gamma^{new} = \left(\sum_{i=1}^{N}\sum_{t=2}^{T} diag\{\left(E_s\left[\boldsymbol{c}_t^{(i)}\boldsymbol{c}_{t-1}^{(i)'}\right]\}-diag\{AE_s\left[\boldsymbol{z}_t^{(i)}\boldsymbol{c}_{t-1}^{(i)'}\right]\}-diag\{\boldsymbol{b}E_s\left[\boldsymbol{c}_{t-1}^{(i)'}\right]\}\right)\right)\left(\sum_{i=1}^{N}\sum_{t=2}^{T} diag\{(E_s\left[\boldsymbol{c}_{t-1}^{(i)}\boldsymbol{c}_{t-1}^{(i)'}\right]\}\right)^{-1}
$$

$$
A^{new} = \left(\sum_{i=1}^{N}\sum_{t=2}^{T}\left(E_s\left[\boldsymbol{c}_t^{(i)}\boldsymbol{z}_t^{(i)'}\right]-\Gamma E_s\left[\boldsymbol{c}_{t-1}^{(i)}\boldsymbol{z}_t^{(i)'}\right]-\boldsymbol{b}E_s\left[\boldsymbol{z}_t^{(i)'}\right]\right)\right)\left(\sum_{i=1}^{N}\sum_{t=2}^{T} E_s\left[\boldsymbol{z}_t^{(i)}\boldsymbol{z}_t^{(i)'}\right]\right)^{-1}
$$

$$\boldsymbol{b}^{new} = \frac{1}{N(T-1)}\sum_{i=1}^{N}\sum_{t=2}^{T}\left(E_s\left[\boldsymbol{c}_t^{(i)}\right] - \Gamma E_s\left[\boldsymbol{c}_{t-1}^{(i)}\right] - AE_s\left[\boldsymbol{z}_t^{(i)}\right]\right)$$

$$\begin{aligned}Q^{new} =&\frac{1}{N(T-1)}\sum_{i=1}^{N}\sum_{t=2}^{T}\Big(diag\{E_s\left[\boldsymbol{c}_t^{(i)}\boldsymbol{c}_t^{(i)'}\right]\} - 2diag\{\Gamma E_s\left[\boldsymbol{c}_{t-1}^{(i)}\boldsymbol{c}_t^{(i)'}\right]\} - 2diag\{AE_s\left[\boldsymbol{z}_t^{(i)}\boldsymbol{c}_t^{(i)'}\right]\}\\ &-2diag\{\boldsymbol{b}E_s\left[\boldsymbol{c}_t^{(i)'}\right]\} + 2diag\{AE_s\left[\boldsymbol{z}_t^{(i)}\boldsymbol{c}_{t-1}^{(i)'}\right]\Gamma'\} + diag\{\Gamma E_s\left[\boldsymbol{c}_{t-1}^{(i)}\boldsymbol{c}_{t-1}^{(i)'}\right]\Gamma'\} + 2diag\{\boldsymbol{b}E_s\left[\boldsymbol{c}_{t-1}^{(i)'}\right]\Gamma'\}\\ &+diag\{AE_s\left[\boldsymbol{z}_t^{(i)}\boldsymbol{z}_t^{(i)'}\right]A'\} + 2diag\{\boldsymbol{b}E_s\left[\boldsymbol{z}_t^{(i)'}\right]A'\} + diag\{\boldsymbol{b}\boldsymbol{b}'\}\Big)\end{aligned}$$

$$D^{new} = \left(\sum_{i=1}^{N}\sum_{t=3}^{T}\left(diag\{E_s\left[\boldsymbol{z}_t^{(i)}\boldsymbol{z}_{t-1}^{(i)'}\right]\}\right)\right)\left(\sum_{i=1}^{N}\sum_{t=3}^{T} diag\{E_s\left[\boldsymbol{z}_{t-1}^{(i)}\boldsymbol{z}_{t-1}^{(i)'}\right]\}\right)^{-1}$$

$$P^{new} =\frac{1}{N(T-2)}\sum_{i=1}^{N}\sum_{t=3}^{T}\left(diag\{E_s\left[\boldsymbol{z}_t^{(i)}\boldsymbol{z}_t^{(i)'}\right]\} - 2diag\{DE_s\left[\boldsymbol{z}_{t-1}^{(i)}\boldsymbol{z}_t^{(i)'}\right]\} + diag\{DE_s\left[\boldsymbol{z}_{t-1}^{(i)}\boldsymbol{z}_{t-1}^{(i)'}\right]D'\}\right)$$

$$\boldsymbol{\mu}_1^{new} = \frac{1}{N}\sum_{i=1}^{N}E_s\left[\boldsymbol{c}_1^{(i)}\right]$$

$$V_1^{new} = \frac{1}{N}\sum_{i=1}^{N}Var\left[\boldsymbol{c}_1^{(i)}\right] + \frac{1}{N}\sum_{i=1}^{N}\left(E_s\left[\boldsymbol{c}_1^{(i)}\right] - \boldsymbol{\mu}_1\right)\left(E_s\left[\boldsymbol{c}_1^{(i)}\right] - \boldsymbol{\mu}_1\right)'$$

$$\boldsymbol{h}_2^{new} = \frac{1}{N}\sum_{i=1}^{N}E_s\left[\boldsymbol{z}_2^{(i)}\right]$$

$$G_2^{new} = \frac{1}{N}\sum_{i=1}^{N}Var\left[\boldsymbol{z}_2^{(i)}\right] + \frac{1}{N}\sum_{i=1}^{N}\left(E_s\left[\boldsymbol{z}_2^{(i)}\right] - \boldsymbol{h}_2\right)\left(E_s\left[\boldsymbol{z}_2^{(i)}\right] - \boldsymbol{h}_2\right)'$$
